# Enriching drought resistance in *Solanum lycopersicum* using Abscisic acid as drought enhancer derived from *Lygodium japonicum*: A new-fangled computational approach

**DOI:** 10.3389/fpls.2023.1106857

**Published:** 2023-02-02

**Authors:** Kahkashan Perveen, Alanoud T. Alfagham, Sandip Debnath, Najat A. Bukhari, Dong-Qing Wei, Najla A. Alshaikh, Aisha Saleh Alwadai

**Affiliations:** ^1^ Department of Botany & Microbiology, College of Science, King Saud University, Riyadh, Saudi Arabia; ^2^ Department of Genetics and Plant Breeding, Institute of Agriculture, Visva-Bharati University, Sriniketan, West Bengal, India; ^3^ Tianjin Institute of Bioinformatics and Drug Discovery (TIBDD), Tianjin Normal University, Tianjin, China

**Keywords:** phytochemicals, drought, MD simulation, molecular docking, drought tolerant, *Solanum lycopersicum*, Abscisic acid

## Abstract

**Introduction:**

Drought is the largest abiotic factor impacting agriculture. Plants are challenged by both natural and artificial stressors because they are immobile. To produce drought-resistant plants, we need to know how plants react to drought. A largescale proteome study of leaf and root tissue found drought-responsive proteins. Tomato as a vegetable is grown worldwide. Agricultural biotechnology focuses on creating drought-resistant cultivars. This is important because tomato drought is so widespread. Breeders have worked to improve tomato quality, production, and stress resistance. Conventional breeding approaches have only increased drought tolerance because of drought’s complexity. Many studies have examined how tomatoes handle drought. With genomics, transcriptomics, proteomics, metabolomics, and modern sequencing technologies, it’s easier to find drought-responsive genes.

**Method:**

Biotechnology and in silico studies has helped demonstrate the function of drought-sensitive genes and generate drought-resistant plant types. The latest tomato genome editing technology is another. WRKY genes are plant transcription factors. They help plants grow and respond to both natural and artificial stimuli. To make plants that can handle stress, we need to know how WRKY-proteins, which are transcription factors, work with other proteins and ligands in plant cells by molecular docking and modeling study.

**Result:**

Abscisic acid, a plant hormone generated in stressed roots, was used here to make plants drought-resistant. Abscisic acid binds WRKY with binding affinity -7.4kcal/mol and inhibitory concentration (Ki) 0.12 microM.

**Discussion:**

This study aims to modulate the transcription factor so plants can handle drought and stress better. Therefore, polyphenols found to make Solanum lycopersicum more drought-tolerant.

## Introduction

Crop production is confronted with a tremendous encounter in the shape of stress, salt and drought, two climate change effects that are particularly harmful to sustainable agriculture. The atmosphere, anticipated to grow substantially less favorable due to ongoing greenhouse gas emissions, would likely limit agricultural production. Evapo-transpiration, dryness, and soil salinity will increase as a result, as will the likelihood of pest and disease infestation. These poor conditions harm plant growth, plant survival, and harvest quality. It is commonly known that potatoes are a crop that suffers tremendously from drought. The fundamental reason is that tomatoes have weaker roots than other plants. When a plant does not get enough water, it experiences stress, which can lead to the death of leaf tissue. This death often occurs in bands that extend outward from the leaf’s borders. Some individuals may get this confused with late blight. When plants are undergoing rapid development or experiencing other conditions that cause them to lose a greater quantity of water through transpiration, plants are particularly susceptible to the adverse effects of this stress. In these conditions, it may be challenging to formulate a plan that takes advantage of periods when the soil is moderately dry and moist enough. The rate at which water is lost through transpiration is slowed when the environment has a high humidity level. When huge plants are kept in confined containers, it is not straightforward to maintain an adequate soil moisture level. A physiological injury, as opposed to late blight, can be diagnosed by the absence of light green, wilted border tissue around leaf symptoms, the absence of pathogen development, and the absence of stem lesions. Extremely low or high temperatures, protracted droughts or floods, excessive salt in the soil, and other abiotic factors are some of the environmental causes that frequently result in low yields (Taheri et al., 2022). A common vegetable that might suffer from unfavorable conditions is the tomato, scientifically known as *Solanum lycopersicum* (Perez-Alfocea et al., 1996). Because they are nearer non-living factors than tomatoes cultivated in greenhouses, tomatoes grown in open fields are more prone to sustain damage from them, how drought and extreme heat affect crop production, the length of the growing season, and the area that may be used for crop cultivation. A plant’s roots lose a lot of their capacity to transmit water when exposed to freezing temperatures. This thickens the water while also making the crucial membrane in the roots less conductive (Venema et al., 2005). As a result, plants absorb less water, which may cause water stress in the shoots. Thus, tomato leaves begin towards wane, and photosynthesis besides water loss are slackened (Ishiguro and Nakamura, 1994).

**Figure 1:**
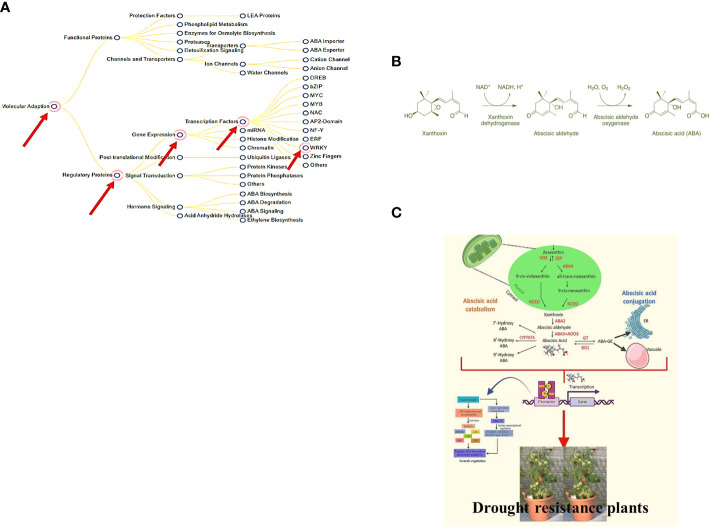
**(A)** Enhanced tolerance to drought & biotic stress and genes responsible (*Available from:* https://pgsb.helmholtz-muenchen.de/droughtdb/drought_db.html). **(B)** Abscisic acid bio-synthesis. **(C)** Role of Abscisic acid in enhancing drought resistance and improved fruits production (Created with Adobe Illustrator).

The WRKY transcription factor family comprehends 5936 members found in various plant systems (PlantTFDB 3.0). WRKY is the DNA-binding protein SPF1 from sweet potato, which may assist control of gene expression, as described for the first time (Ishiguro and Nakamura, 1994). The word “WRKY” denotes to their WRKY domain, which instigates with a relatively constant sequence of WRKYGQK at the N-terminus and ends with a Cx4-5Cx22-23HxH or Cx7Cx23HxC zinc-finger motif (Rushton et al., 1996; Zou et al., 2004). Much study has been conducted on the WRKY domain, mainly how it controls genes and communicates with other molecules within plant cells. WRKY transcription factors were initially assumed to be a part of a plant´s defense against pathogens (Zou et al., 2004; Rushton et al., 2010). However, the subsequent study has revealed that they are related to abiotic stresses (Rushton et al., 2010), seed dormancy and germination (Qiu and Yu, 2009; Akbudak and Filiz, 2020), and seed development (Alexandrova and Conger, 2002; Guo et al., 2004). Most WRKY proteins (Chen and Chen, 2000; Cormack et al., 2002; Zou et al., 2004) can be identified by their capacity to recognize TTGACC/T W-box sequences in the promoter region, although this does not imply that they all perform the same function. So, controlling the specificity of WRKY transcription factors requires more than just identifying the essential W box promoter components. This could be because of interactions with proteins involved in signal transduction, transcription, and chromatin remodeling. Earlier reports demonstrated the role of WRKY transcription factors as negative regulators of abiotic stresses through the constitutive countenance of the BcWRKY46 gene in transgenic tobacco under the CaMV35S promoter, which made the transgenic tobacco more susceptible to cold, abscisic acid (ABA), salt, and dehydration stresses (Xu et al., 2006). There is evidence that WRKY proteins can talk to calmodulin, a signaling molecule that binds Ca^2+^. Recent proteomics research showed that WRKY and 14-3-3 proteins work together  (Xu et al., 2006) (illustrated in **Figure 1A**).

The majority of ABA synthesis (illustrated in **Figure 1B**) takes place in the vascular tissues, and then it is delivered to the target tissues. Because this transport happens in both the xylem and the phloem, it makes it possible for substances to move between the roots and the shoots in either way. The abbreviation ABA was given that name because people used to believe that ABA was a part of the abscission process. Scientists have since discovered that this is only true for a small number of plant species. ABA is also critical to a plant’s defense against environmental stress and disease. It accomplishes this by assisting in the transmission of signals. The order of the steps in the ABA production pathway and the plant genes that produce it have been determined. Some plant-harming fungi can also produce ABA *via* a different biosynthetic pathway than plants (Siewers et al., 2004; Wang et al., 2016). This stops the plant’s growth and instructs the leaf precursors to produce scales to defend the buds while they snooze during the winter. ABA also impedes cell division in the vascular cambium. This permits the plant to reply to cold winter temperatures by halting mutually primary and secondary development (Wang et al., 2016). ABA can also be twisted in plant roots when there is less water in the soil (like when the soil is dry) or when the plant is stressed in some other way. Following that, ABA travels to the leaves, where it rapidly modifies the osmotic potential of stomatal guard cells, shrinking them and sealing the stomata. When there is insufficient water, the ABA causes the stomata to close.

This reduces transpiration and prevents the leaves from losing additional water. Water leaves the plant *via* the stomata during the transpiration process. A robust linear association was discovered between the amount of ABA detected in the leaves and how well the stomata let air in and out based on leaf area (stomatal resistance) (Steuer et al., 1988). The fern species Lygodium japonicum is known by the common names like vine fern (Bradley, 2022) and Japanese climbing fern. It is indigenous to eastern Asia, including Taiwan, Japan, Korea, southeast Asia, and India, as well as eastern Australia. The fern is an invasive species in the southeastern United States and Puerto Rico (Munger, 2005). This plant is harvested for its abscisic acid content (Ohishi et al., 2021). ABA is a hormone that has been around for a long time. It dates back to the first living things, probably the ancestors of modern (ABA-producing) cyanobacteria, long before the plant and animal kingdoms were split. It has always worked as a signal that controls how cells respond to environmental changes. Nanomolar ABA controls how the metabolism of mammals responds to the availability of glucose by increasing energy expenditure in brown and white adipose tissue and turning on glucose uptake in skeletal muscle and adipose tissue in a way that doesn’t require insulin. In contrast to insulin’s activation of AMPK-inhibiting Akt, ABA’s activation of AMP-dependent kinase (AMPK) causes white adipocytes to turn brown and increases muscle glucose uptake through GLUT4. People at risk for prediabetes and metabolic syndrome can improve their blood glucose, lipids, and morphometric parameters (waist circumference and body mass index) with a chronic dose of this amount of ABA. When people with standard or borderline glucose tolerance take in micrograms of ABA per kg of body weight, their glucose tolerance improves (Magnone et al., 2020) (–).-abscisic acid is the unnaturally occurring (1’R)- (-) enantiomer of abscisic acid, it functions as a hormone for plants. This substance is an enantiomer of (+)-abscisic acid (Ohishi et al., 2021). We found Abscisic acid as a product from plant self-hormone which shows a promising result in drought and plant stress tolerance.

Molecular docking is the most commonly applied technique in structure-based ligand design since it allows one to consider the action of small molecules in selected protein targets and predict the structure of the ligand-receptor complex (Meng et al., 2011; Ferreira et al., 2015). This study aimed to find a new compound (Abscisic acid) in the PubChem database that worked well against transcription factor in WRKY gene in enhancing drought resistance. Virtual screening and a structure-based docking approach were used to find the best compound for effective target inhibition. With the help of molecular dynamics simulation studies, this study also looked at the structural stability of the target compound and the best compound. The overall plan is to find a new compound that is a robust drought tolerance enhancer in *Solanum lycopersicum* to be used as a potential candidate against drought resistance (as illustrated in **Figure 1C**).

## Resources and methods

### Potential target preparation

Accessible from the RCSB protein data bank (PDB) (https://www.rcsb.org/2AYD) are 3D structures of the β-Secretase protein for study. The structure stayedexported using a freely accessible molecular editor (Discovery studio visualizer 4.0). Co-crystal ligands and heteroatoms were deleted before the structure was saved in.pdb format. The Chimera UCSF team employed a thousand-step steepest-descent and a thousand-step Conjugate gradient of energy minimization approach for this optimization. Abscisic acid (**ChemI. D: 643732**) was downloaded as a.sdf file from PubChem. National Center for Biotechnology Information (2023). These.pdb files result from a ligand structure loaded into the Discovery Studio visualizer.

### Virtual screening of selected compounds

The active site of an enzyme is a region of an enzyme that possesses an explicit profile that makes it possible for it to connect with a certain molecular substrate (Jorgensen et al., 1996; Mukerjee et al., 2022). This binding then results in a chemical reaction that is caused by the enzyme. An enzyme’s active site is a section of an enzyme that possesses a particular shape that enables the enzyme to bind with a particular molecular substrate. An active site can be described as a portion of an enzyme that contains this shape. AS ensures that the catalytic microenvironments are ideal and acceptable, and it helps chemical compounds develop adequate contact sites so that they can generate strong binding with the enzymes of choice. This allows the chemical compounds to catalyze reactions more effectively. Therefore, in order to establish a high binding affinity with our chemical, the active side of the protein was evaluated using BIOVIA Discovery Studio Visualizer v19.1.0.18287. This was done so that we could achieve our goal. In order to get a high degree of binding affinity, this step was taken. Utilizing the AutoDockvina, virtual screening tool allowed for the determination of the protein’s binding site, which was then employed in the process of constructing the receptor grid. The virtual high throughput screening of 10 different compounds was carried out with the help of AutoDock vina 4.2.6. The compounds were chosen based on the best binding energy scores they achieved with the macromolecule with PDB ID: 2AYD. The best docked posture with the highest binding energies was chosen for re-docking and additional research out of the nine possible poses for each ligand, which were ranked from top to lowest according to the binding energies they produced.

### Molecular docking studies

In the AutoDock MGL tool 1.5.6, the receptor protein to be utilized for docking was produced. To construct the receptor grid, residues around 2Å the 2AYD were linked to the co-crystal of Abscisic acid were utilized. Receptors and ligands were saved in.pdbqt format for future use with the MGL application, and vina was launched using the command line from a command prompt. The exhaustiveness was set to 8 and the default grid point spacing was 0.431 Å. The.pdbqt output files were processed using PyMol and the Discovery studio visualizer 2021. The co-crystallized ligand was used to validate and optimize the ligand binding.The molecular mechanism of the target protein (PDB ID: 2AYD) that is involved in the binding of Abscisic acid. The purpose of this work is to identify the most effective molecule, as determined by virtual screening, for interacting with 2AYD and to determine the inhibitory concentration of each candidate molecule. The structure of 2AYD was simplified by employing the steepest descent method (1000 steps), which was then followed by the incorporation of the AMBER ff4 force field. This was done before the docking research with the necessary ligands could begin. Before beginning the investigations into the interaction, the protonation states of the 2AYD that would be involved were checked for neutralization. Experiments on molecular docking were carried out with the help of AutoDock version 4.2.6 (Steuer et al., 1988; Bowers et al., 2006). Polar hydrogen bonds, Kollman and Gastieger charges, and other electrostatic forces were combined to produce not just the receptor but also the ligands. After merging the nonpolar hydrogens, the receptor and ligand molecules were finally saved in the.pdbqt format. With the values X=12, Y=20, and Z=30, with a spacing of 0.33 angstrom, a grid box was produced. Lamarckian Genetic Algorithm was used to dock protein-ligand complexes to get the lowest binding free energy (ΔG).

### Molecular dynamic Simulations

Schrodinger, LLC’s Desmond 2020.1 was utilized to conduct 100 ns MD simulations of the main protein, 2AYD, along with the ligand Abscisic acid. The explicit solvent model with SPC water molecules and the OPLS-2005 force field were utilized in this particular system (Martyna et al., 1992; Martyna et al., 1994; Shivakumar et al., 2010). To eliminate the charge, multiple Na+ ions were administered. In order to simulate the physiological environment, 0.15M NaCl solutions were added to the system. In each simulation, the NPT ensemble was generated by applying the Nose-Hoover chain coupling method (Goodsell et al., 1996; Ghosh et al., 2022). The simulations were conducted with the following parameters: a temperature of 300 K, a relaxation period of 1.0 ps, a pressure of 1 bar, and a subsequent time step of 2 ps. The Martyna–Tuckerman–Klein chain coupling system (Toukmaji and Board, 1996) barostat method was used, and the relaxation time was set to 2 picoseconds. This method was used to regulate the pressure. To predict long-range electrostatic interactions, the Colombian interaction radius was set to 9 and the particle mesh Ewald technique (Bowers et al., 2006) was employed. Utilizing the RESPA integrator, the unbonded forces were determined. The root mean square deviation was utilized to assess the ability of MD simulations to maintain stability.

## Results

### Virtual screening of compounds

Binding empathy of the complex, 2AYD-643732 posses-7.4kcal/mol score. The most promising molecule underwent additional reassembly in the binding cavity of 2AYD. A total of 10 ligands were investigated as potential binding partners for the 2AYD receptor protein shown in **Table 1**. This virtual screening based on binding energy gave us a vivid idea of best ligand having highest affinity with the targeted protein.

**Table 1:** Molecular Docking of 10 selected phyto-compounds.

Sl No.	Compound ID (CID)	Binding energy (Kcal/mol)
1.	CID_100332	-5.6
2.	CID_101389368	-5.5
3.	CID_119034	-6.4
4.	CID_15559069	-7.1
5.	CID_241572	-7.0
**6.**	**CID_643732**	**-7.4**
7.	CID_3981577	-6.9
8.	CID_5280343	-5.3
9.	CID_5280443	-7.0
10.	CID_5280445	-6.2

Ligand bound to the core of the pocket with lowest binding energy is indicated in bold.

### Molecular docking investigation

Molecular docking is a method that can be employed to ascertain the intermolecular framework that is optimally shaped by a macromolecule in conjunction with medication or added small molecular contender. At the outset, molecular docking research was conducted to screen for and locate the intermolecular interaction that would be most beneficial between the protein of interest and the phytochemical substances. PyRx instruments: To carry out molecular docking between 10 phytochemical compounds with a three-dimensional structure and desire protein, the AutoDock vina wizard has been used. **Table 1** shows the binding affinities of 10 different phytochemicals. Molecular interaction in re-docking studies of ligand Abscisic acid with 2AYD displayed well defined binding pocket constituted of residues leucine, valine, proline, alanine, asparagine, serine, cystine, leucine, glycine where the ligand bound to the core of the pocket with binding energy (ΔG**)**-7.4kcal/mol and inhibitory concentration (Ki) 0.12microM.

### Molecular dynamic simulation study

**Figure 2:**
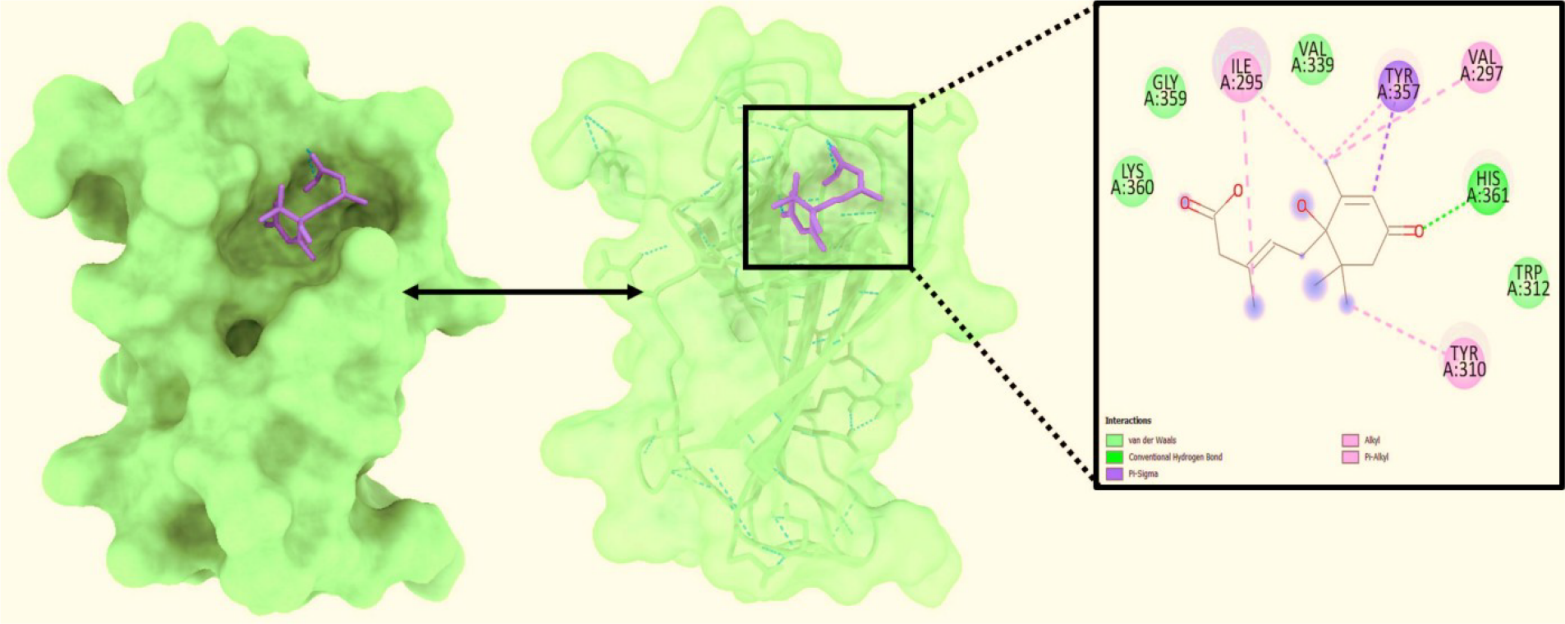
Analysis of the docked posture of 2AYD-643732; were displayed the ligand bound at the pocket of the receptor 2AYD and the binding pocket residues interacted with the ligand displayed.

**Figure 3:**
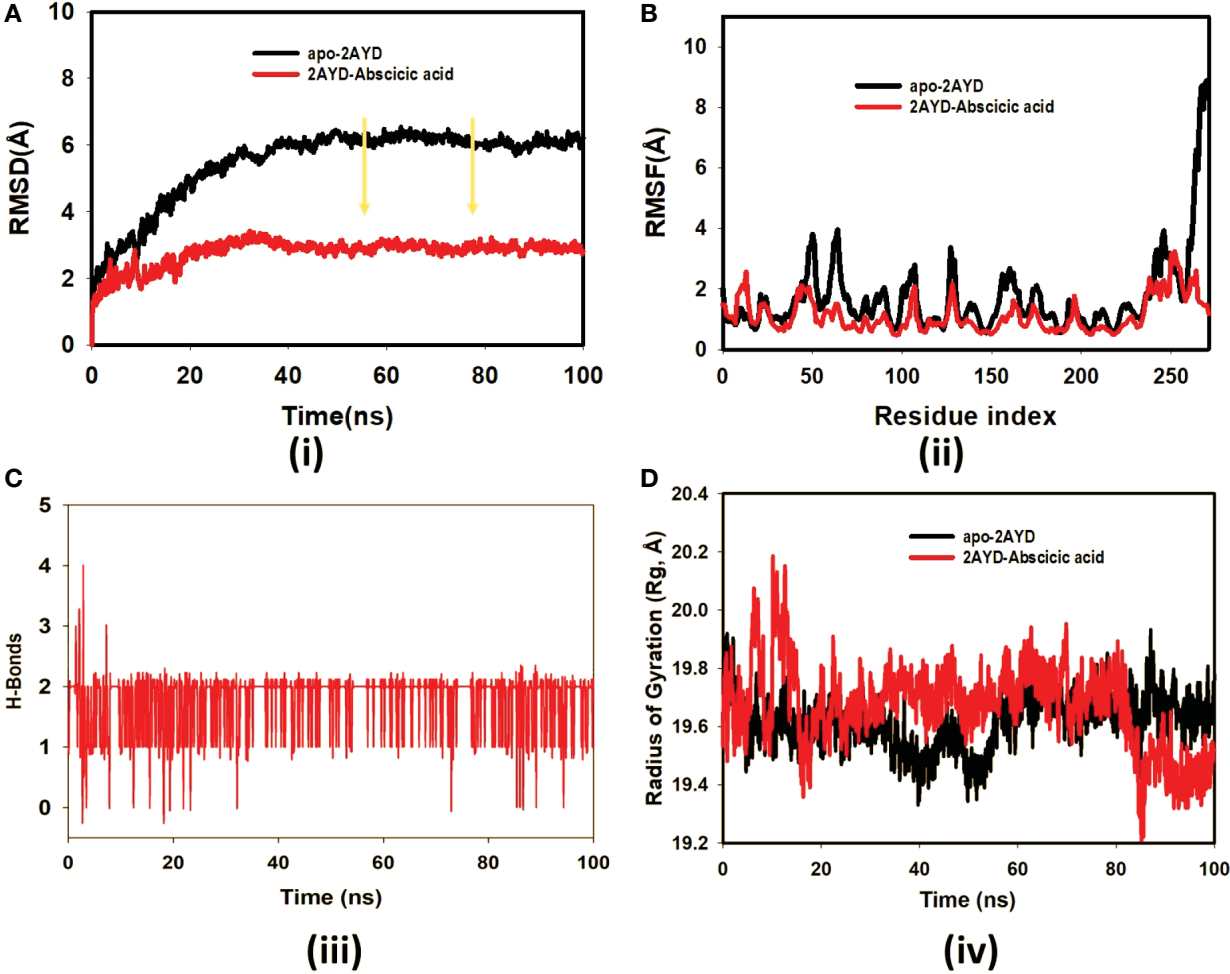
**(A)** RMSD of 2AYD and ligand Abscisic acid for 100 ns; **(B)** RMSF of 2AYD and ligand Abscisic acid for 100 ns; **(C)** Number of Hydrogen bindings of 2AYD and ligand Abscisic acid for 100 ns; **(D)** Radius of gyration of 2AYD and ligand Abscisic acid for 100 ns.

The ligand Abscisic acid (Chem ID:643732) and the 2AYD protein were subjected to MD simulation studies for one hundred nanoseconds to examine the overall quality and stability of the complex till convergence. The root means square deviation (RMSD) of the C-α, the backbone of 2AYD (depicted in **Figure 2**) with ligand coupled complex, revealed an extremely stable structure, with a fluctuation of only 1.99Å. On the other hand, the RMSD of the ligand Abscisic acid was slightly distorted in the beginning up. However, it remained stable until 100 ns without any significant variations (depicted in **Figure 3**. i). On the other hand, the root means square fluctuations (RMSF) of the different amino acids that make up the C-α backbone of 2AYD showed the most negligible fluctuations, which indicates that the protein structure is stable. The relative mean squared deviation of Abscisic acid-bound protein simulation trajectories across a timescale of 100 ns. Every piece of data is measured three times to ensure accuracy, and the Y-axis is shifted after each iteration. After a total of 100 ns, the final structure of 2AYD had significant deviations from the reference structure, with an average difference of 2 between it and residue positions 230-250. (depicted in **Figure 3**. ii). **Figure 3**. iii provides a visual representation of the typical number of hydrogen bonds established between Abscisic acid and the various proteins throughout the 100 ns simulation. The MD simulation of Abscisic acid and 2AYD revealed a significant number of hydrogen bonds. Throughout the simulation, a total of two hydrogen bonds were established. The increased number of hydrogen bonds between protein 2AYD and Abscisic acid has helped to strengthen the binding and enhancing the drought resistance, which has contributed to the simulation’s success in maintaining its stability. In addition, the radius of gyration, also known as Rg, was calculated. Rg is an indicator of the size and compactness of the protein when it is in a state where it is attached to the ligand. **Figure 3**.iv depicts the Rg plots for the convenience. According to the Rg plot of the C-α backbone, the 2AYD protein displays Rg values ranging from 27.8 to 28.0 angstroms, suggesting significant compactness with an average of 0.3 angstroms from the start to the finish of the 100 ns simulation.

**Figure 4:**
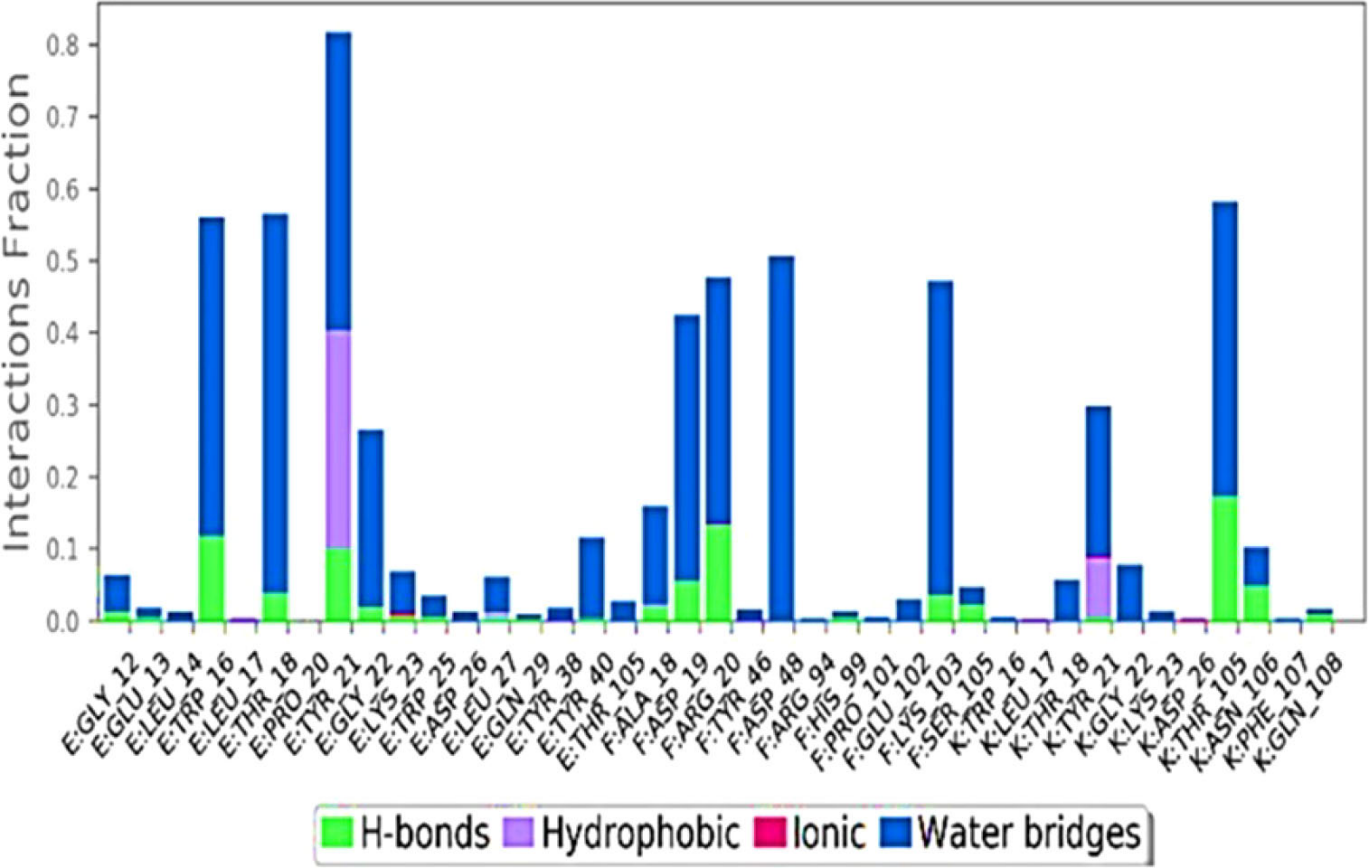
Types of bond formed in 100ns simulation run.

Ligand interaction of Abscisic acid with predicteddocked residues of 2AYDdemonstratedtheestablishment of substantial hydrogen bonds and apart from this, other non-bonded interactions such as hydrophobic interaction as well as water bridges (illustrated in **Figure 4**). These interactions interplayed critical role in making a stable complex between the protein and the ligand.

**Figure 5:**
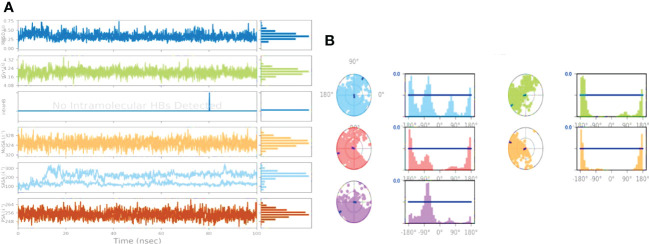
**(A)** The figure displayed shows ligand characteristics such as RMSD, RoG, Hydrogen bonding, MolSA, SASA, and polar surface area (PSA) of Abscisic acid; **(B)** Ligand torsion profile after 100 ns simulation.

The illustration in **Figure 5A** highlights ligand characteristic such as RMSD, radius of gyration (rGyr), intra-molecular hydrogen bond, molecular surface area (MolSA), solvent accessible surface area (SASA), and polar surface area (PSA). In the ligand, no intra-molecular hydrogen was found. In **Figure 5B**, ligand torsion map shows the structural evolution of each rotatable bond (RB) over time (0.00 through 100.00 ns). Top: Two-dimensional graphic illustrating rotatable ligand linkages. Dial plots and bar plots, both the same color, indicate rotatable bonds. As the simulation progresses, dial (or radial) charts depict the torsion’s conformation. The simulation is traced in a circle from the display’s center. Dial plots and bar charts depict the torsion’s probability distribution. Infographic shows rotatable bond strength (if torsional potential information is given). Check the chart’s left Y-axis for available values. When conducting this type of research, it’s crucial to monitor the histogram, torsion potential, and protein’s conformation strain to evaluate if the bound shape is maintained.

## Discussion

Drought severity is the abiotic factor with the most significant impact on agricultural output. Because plants do not migrate from their fixed positions, they are vulnerable to natural and artificial pressures. More understanding of how plants react to drought is needed to devise breeding procedures that will make plants more drought-resistant. A thorough analysis of the proteome of leaf and root tissue revealed the presence of proteins that respond to drought in several unique ways depending on the genotype of the plant. WRKY genes are members of one of the most diverse plants transcription factor families, and they play an essential part in how plants respond to both natural and unnatural stimuli and how they grow and develop as a whole.

**Figure 6:**
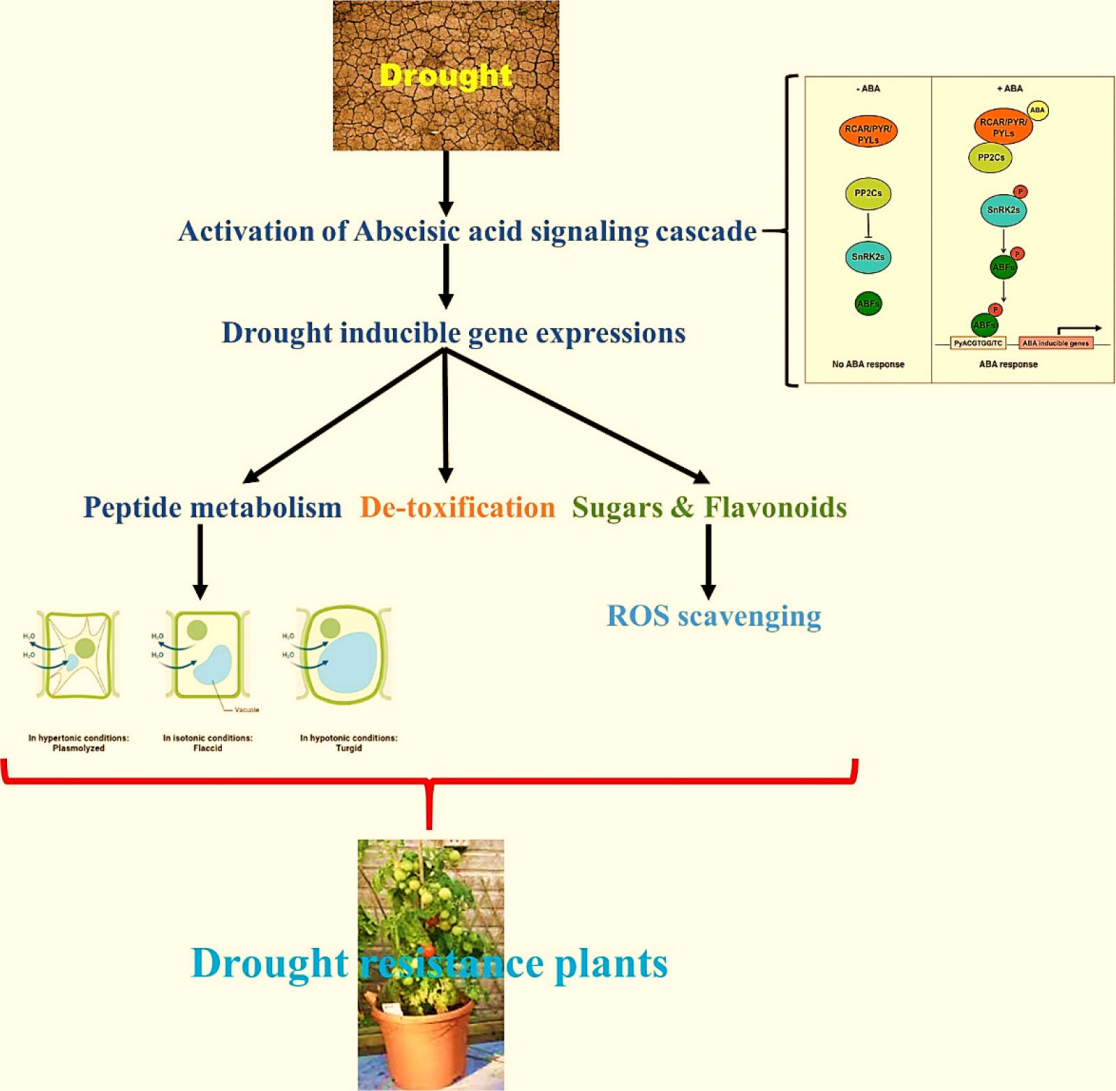
Research outcome in using Abscisic acid as drought resistance enhancer factor.

Furthermore, WRKY genes influence how plants respond to natural and manmade stimuli. Understanding how WRKY proteins interact with other proteins and ligands in plant cells is critical to creating plants that can withstand biotic and abiotic stressors. The utilization of abscisic acid, a hormone produced in plant roots when stressed, is the focus of this research endeavour, which aims to make plants more drought-resistant and stress resistant by modulating the transcription factor (as illustrated in **Figure 6**).

## Conclusion

Through molecular dynamics simulation, we investigated the expected structure’s peculiar responses when placed in an aqueous environment. In addition, the structure can be used to evaluate the complexity of any ligand or molecule designed to interact with the WRKY transcription protein possessing a binding affinity of -7.4 kcal/mol. Abscisic acid has a high affinity for the WRKY protein. These polyphenols are a powerful component that, according to a study that used molecular docking and modeling, makes *Solanum lycopersicum* more drought tolerant. The binding energies of the ligand-target interaction are vital for characterizing the affinity with which the drug binds to the drought transcription factor-associated target. Finally, we are able to identify Abscisic acid as a potential phytoagent which can enhance drought tolerance in tomato plants.

## Data availability statement

The original contributions presented in the study are included in the article/supplementary material. Further inquiries can be directed to the corresponding authors.

## Author contributions

KP, AA and SD: Conceptualization, designing experiment, Investigation, Data analysis, Supervision, Manuscript writing and editing. NB: Designing experiment, Data collection and analysis, Manuscript writing and editing. D-QW, NA and AA: Manuscript writing and revision editing. All authors contributed to the article and approved the submitted version.
